# Squamous metaplasia of the *rete ovarii* in a Zebu cow

**DOI:** 10.1186/1746-6148-8-235

**Published:** 2012-12-05

**Authors:** Renato Lima Santos, Dimitre Giancarlo de Medeiros Peixoto, Andréia Pereira Turchetti, Álan Maia Borges, Ernane Fagundes do Nascimento, Tatiane Alves Paixão

**Affiliations:** 1 Departamento de Clínica e Cirurgia Veterinárias, Escola de Veterinária, Universidade Federal de Minas Gerais, Minas Gerais, Brasil; 2 Departamento de Patologia Geral, Instituto de Ciências Biológicas, Universidade Federal de Minas Gerais, Minas Gerais, Brasil

**Keywords:** Ovary, Cow, *Rete ovarii*, Squamous metaplasia, Epidermoid cyst

## Abstract

**Background:**

Stratified keratinizing squamous epithelium in the ovary has been associated with the diagnosis of ovarian teratoma in cows. Recently, the diagnosis of “epidermoid cyst” has been proposed. A case of squamous metaplasia of the *rete ovarii* in a Zebu cow is described in this report.

**Case presentation:**

A crossbreed Zebu cow had both ovaries enlarged with multiple cysts. Most cysts were lined by well differentiated keratinizing stratified squamous epithelium and filled with keratinized lamellar material. Some cysts were lined by an epithelial layer that ranged from single cuboidal, double cuboidal epithelium, stratified non keratinized epithelium, and areas of keratinizing stratified squamous epithelium. Single or double layered cuboidal epithelia of the cysts expressed low molecular weight cytokeratin 7, whose expression was absent in the keratinizing stratified squamous epithelia of same cysts. Conversely, high molecular weight cytokeratins 1, 5, 10, and 14 were strongly expressed by the keratinizing stratified epithelium.

**Conclusion:**

Squamous metaplasia of the *rete ovarii* was diagnosed. Squamous metaplasia of the *rete ovarii*, may account for some of the previously described squamous lesions in the ovary, which may have been misinterpreted as teratoma or epidermoid cysts.

## Background

Stratified keratinizing squamous epithelium in the ovary has been historically associated with the diagnosis of ovarian teratoma in cows [1,2]. McEntee (1990) [1] has even used the terminology “ovarian dermoid cyst” as a synonym of benign ovarian teratoma, mentioning that this lesion is more common in Zebu cattle [1,3]. More recently, the diagnosis of epidermoid cyst has been proposed for the first time in the veterinary literature to describe ovarian cysts lined by stratified keratinizing squamous epithelium in the absence of hair follicles and adnexal glands [4].

In women, epidermoid cysts have been described, although there are evidences that most epidermoid cysts are in fact insufficiently sampled teratomas, leading some investigators to question the appropriateness of the diagnosis of epidermoid cysts [5]. Ovarian squamous lesions in women are often associated with teratomas, although it may be found as a metaplastic component in ovarian carcinomas or ovarian endometriosis [5,6]. To the best of our knowledge, squamous metaplasia of the *rete ovarii* has not been previously diagnosed in human medicine. This lesion has been previously mentioned, but not described or documented, in a study of follicular cysts and cysts of the corpora lutea in cows [7]. Here we describe a case of squamous metaplasia of the *rete ovarii* in a Zebu cow.

## Case presentation

**Figure 1:**
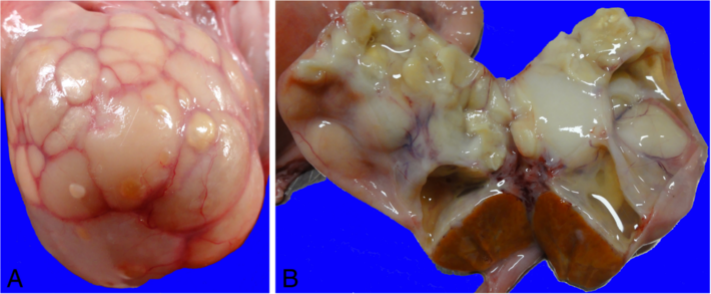
**Gross appearance of a bovine ovary with squamous metaplasia of the *rete ovarii.*** (**A**) Enlarged right ovary with an irregular surface containing multiple cysts. (**B**) Cut surface of the right ovary with multiple cysts and a corpus luteum. Some of the cysts are filled with a translucent or whitish mucous material, while other cysts are filled with a dense keratinized material.

Genital organs including the uterus, uterine tubes, and ovaries from a crossbreed Zebu cow were submitted by a local slaughterhouse for pathological evaluation. Both ovaries were enlarged, measuring approximately 8 × 6 × 5 and 6 × 5 × 5 cm (right and left, respectively). The ovaries had an irregular surface with multiple cysts, most of which with a white to light yellow thick wall, and a few with a thin translucent wall (Figure 1A). These cysts ranged from 0.3 to 5.0 cm in diameter, and most of them were filled with a yellowish dense material while some contained a mucous whitish or transparent content (Figure 1B). In the right ovary, there was an apparently functional corpus luteum, which was solid, homogeneous, and had a dark yellow color on its cut surface. The uterus and uterine tubes had no lesions, although the perimetrium had several longitudinal striations indicating that the cow has been previously pregnant.

Tissue fragments from both ovaries were fixed by immersion in 10% buffered formalin, processed for paraffin embedding. Tissue sections were stained with hematoxylin and eosin, and Masson’s Trichrome. Selected sections were processed for immohistochemistry, using primary antibodies against the low molecular weight cytokeratin 7 (clone OV-TL 12/30, Dako, code M7018, dilution 1:100), heavy molecular weight cytokeratins 1, 5, 10, and 14 (clone 34BE12, Dako, code M0630, dilution 1:40), and vimentin (clone Vim3B4; Dako, code M7020, dilution 1:100), with the streptavidin-biotin-peroxidase complex as detection system (LSAB+ kit, Dako Corporation, Carpinteria, California,USA), and diaminobenzidine (DAB) as chromogen.

**Figure 2:**
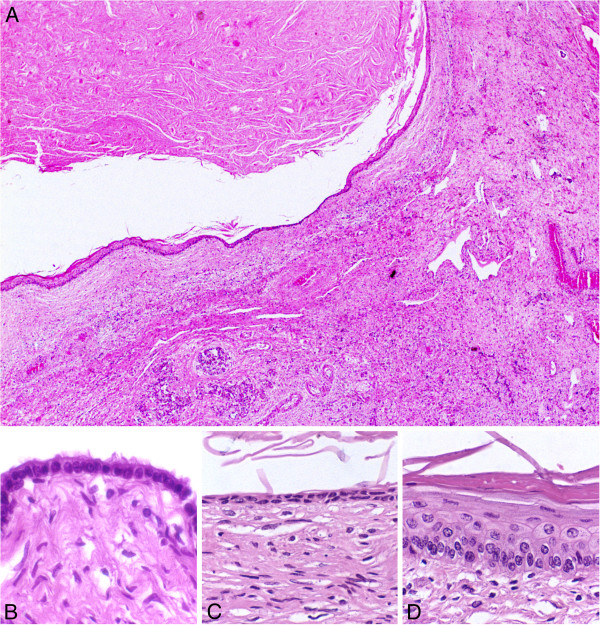
**Microscopic features of a bovine ovary with squamous metaplasia of the *rete ovarii.*** (**A**) Cystic cavity in the ovary filled with keratinized material and lined by single layered cuboidal epithelium (arrowhead) transitioning into a keratinizing stratified squamous epithelium (arrow). (**B**) Detail of the cystic wall with an area of single layered cuboidal epithelium. (**C**) Detail of the cystic wall with an area of double layered epithelium. (**D**) Detail of the cystic wall with an area of keratinizing stratified squamous epithelium. Hematoxylin and eosin.

Histologically, most of the cysts were lined by well differentiated keratinizing stratified squamous epithelium and filled with keratinized lamellar material. Interestingly, some smaller cysts were lined by cuboidal, rarely flagellated, single layered epithelium, supported by connective tissue that was interpreted as cystic *rete ovarii*. However, these cysts contained sparse keratinized material. Some other cysts were lined by an epithelial layer that ranged from single cuboidal (Figure 2A and B), transitioning into double cuboidal (Figure 2C), then into a stratified non keratinized, and finally into an area of keratinizing stratified squamous epithelium (Figure 2A and D). These were also filled with keratinized material. Absence of smooth muscle cells in the wall of these cysts was observed in hematoxylin and eosin stained sections, and further confirmed by Masson’s trichrome staining.

**Figure 3:**
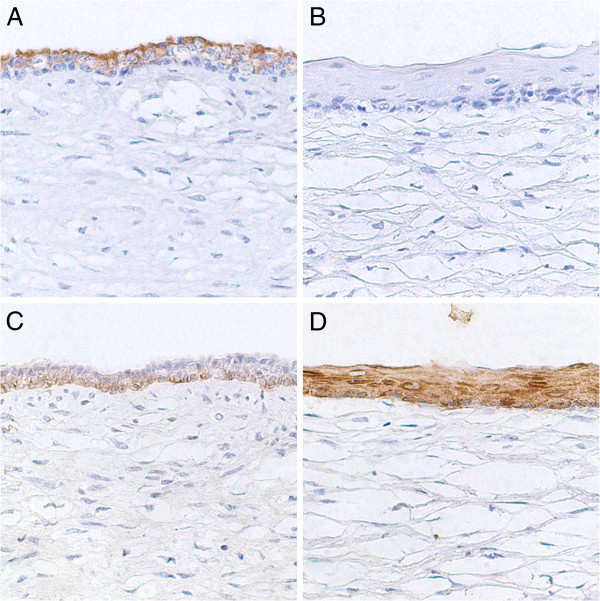
**Immunohistochemical features of a bovine ovary with squamous metaplasia of the *rete ovarii.*** (**A**) Expression of low molecular weight cytokeratin 7 (clone OV-TL 12/30) in an area of double layered cuboidal non keratinized epithelium. (**B**) Ovary, cow. Absence of expression of low molecular weight cytokeratin 7 (clone OV-TL 12/30) in an area of keratinizing stratified squamous epithelium. (**C**) Low levels of expression of high molecular weight cytokeratins 1, 5, 10, and 14 (clone 34BE12) in an area of double layered cuboidal non keratinized epithelium. (**D**) Strong expression of high molecular weight cytokeratins 1, 5, 10, and 14 (clone 34BE12) in an area of keratinizing stratified squamous epithelium. Streptavidin-biotin-peroxidase, Hematoxylin counterstain.

Immunohistochemistry demonstrated that the single or double layered cuboidal epithelia of the cysts expressed low molecular weight cytokeratin 7 (Figure 3A), whose expression was absent in the keratinizing stratified squamous epithelia (Figure 3B). Conversely, the single or double layered epithelia had low levels of expression of high molecular weight cytokeratins 1, 5, 10, and 14 (Figure 3C), whereas the keratinizing stratified epithelium strongly expressed these cytokeratins (Figure 3D). As expected, epithelial cells did not express vimentin that was strongly expressed by stromal cells of the connective tissue.

Here we describe a case of squamous metaplasia of the *rete ovarii* in a Zebu cow. Although this is the first detailed description of this lesion, it has been previously mentioned in a study that was aimed to evaluate cystic corpora lutea and cystic follicles in cows, which mentioned, but not documented, two cases of squamous metaplasia of the *rete ovarii*[7]. However, Edwards (2002) [4] described that in one of three cases diagnosed as epidermoid cysts, the cyst was lined by cuboidal occasionally ciliated epithelium compatible with the epithelium of the *rete ovarii*. Furthermore, reevaluating the work by Costa (1974) [3], there was clear description and illustration of lesions that have been interpreted as teratomas, that were characterized by cysts filled with keratinized material and lined predominantly by a keratinizing stratified epithelium that contained areas with cuboidal rarely ciliated epithelium, and therefore, morphologically indistinguishable from the case reported here. In addition, McEntee (1990) [1] described “ovarian dermoid cyst” as a synonym of benign ovarian teratoma. Thus, our findings along with these previously reported studies support the notion that very likely some of the lesions previously diagnosed as teratoma or epidermoid cysts may in fact represent cases of squamous metaplasia of the *rete ovarii*.

Absence of vimentin expression by the cuboidal epithelial cells in this case is consistent with a previous study, which reported that vimentin is expressed by the epithelium of the *rete ovarii* in only half of the human ovaries examined [8].

All findings described in this case strongly support the interpretation that this is not a neoplastic lesion, which is quite different from the pathogenesis of ovarian teratomas [1,2]. Furthermore, a congenital nature has been proposed in cases interpreted as epidermoid cysts [4], but the absence of reported cases in neonatal or young calves does not support this hypothesis.

In spite of cystic changes occupying extensive areas in both ovaries, the cow in this case was still capable of ovarian cyclic activity, as evidenced by a mature and apparently functional corpus luteum in the right ovary. Furthermore, this particular cow had been previously pregnant as evidenced by multiple longitudinal striations in the perimetrium, suggesting that the squamous metaplasia of the *rete ovarii* tends not to prevent ovarian cyclicity and pregnancy.

## Conclusion

In conclusion, here we describe a case of squamous metaplasia of the *rete ovarii* that in spite of its low frequency (or rarity) may account for some of the previously described squamous lesions in the bovine ovary, which likely may have been misinterpreted as teratoma or epidermoid cysts.

## Competing interests

Authors declare that they have no competing interests.

## Authors’ contributions

DGMP and AMB performed slaughterhouses surveys that resulted in identification of this case. RLS and RFN performed gross and histopathology that diagnosed the case. APT and TAP performed immunohistochemistry. RLS wrote drafted the manuscript. DGMP, APT, EFN, and TAP critically reviewed the manuscript. All authors read and approved the final manuscript.
